# Competition in health research: the experience of the John Curtin School of Medical Research

**DOI:** 10.1186/1743-8462-2-5

**Published:** 2005-03-16

**Authors:** Judith A Whitworth

**Affiliations:** 1 John Curtin School of Medical Research, Australian National University, Canberra ACT 0200, Australia

## Abstract

**Background:**

In 2002 the Australian National Competitive Grants System was opened to the Institute of Advanced Studies at the Australian National University as part of a commitment to transparency, competitiveness, and collaboration in national research funding.

**Results:**

The block grant to the John Curtin School of Medical Research had progressively eroded over many years. Access to the National Competitive Grants Schemes and associated infrastructure (through an agreed 'buy-in' price of 20% of block funding) has succeeded in its aims and in reversing this progressive effective decrease in funding.

**Conclusion:**

Access to the National Competitive Grant Scheme has allowed the John Curtin School of Medical Research to contribute more broadly to Australia's health and medical research effort through increased collaboration, in a transparent and competitive funding environment.

## Discussion

Australia has developed a unique blend of medical research institutions, from universities, teaching hospitals, independent medical research institutes to a range of smaller centres. Universities with medical schools have been the primary forces in medical research and many of the university teaching hospitals fostered the development of research institutes within their grounds [1]. The spectacular development of medical research in Australia over the last 50 years was reviewed recently in recognition of Australia's Centenary of Federation celebrations [2].

The Australian National University (ANU) was established as a research university [1] with four founding schools. The John Curtin School of Medical Research (JCSMR) was funded for its first 50 years through a one-line Commonwealth Grant to the ANU. Although the School had always sought external funds (its first grant was £1500 to Frank Fenner from the Rural Credits Development Fund in 1952), it was ineligible for National Health and Medical Research Council (NHMRC), Australian Research Council (ARC) and other national competitive grants. The School unquestionably benefited from generous (by local standards) funding in its early years and its science profited accordingly. Fundamental discoveries have included Nobel Prize winning work on the elucidation of mechanisms of transmission of signals in the nervous system (Eccles) and the discovery of the role of the major histocompatibility complex (Doherty and Zinkernagel). Today, the School has a wide range of research in such areas as infection, neurosciences, genomics and molecular bioscience, and has spawned a major national research facility, the Australian Phenomics Facility. Some key discoveries in recent years are summarised in Table 1.

**Table 1:** Recent Research Achievements, John Curtin School of Medical Research

**Year**	**Achievement**	**Researchers(s)**	**Comment**	**Reference**
1999	Heparanase cloned	Hulett, Freeman and colleagues	Inhibiting the enzyme is the basis of cancer treatments based on sulfated polysaccharides	Nature Medicine 5:803-809, 1999
2001	First diabetes susceptibility gene identified	Slattery and colleagues	Serendipitous discovery providing a potential therapeutic target in type 1 diabetes	Proc Nat Acad Sci, USA 98:11533-11538, 2001
2002	Amiloride derivatives block ion channel activity and enhancement of virus-like particle budding caused by an HIV-1 protein	Gage and colleagues	Research on compounds that block viral ion channels raises the possibility of inhibiting viruses that utilize ion channels	Eur. Biophys. J. 31:26-35, 2002
2002	Antibody 'tail sequence' identified that has significant implications for immunological memory	Martin and Goodnow	Finding has wide implications for vaccination, allergy and autoimmunity	Nature Immunology 3:182-188, 2002
2002	Phase II clinical trials of PI 88 anti-cancer drug	Parish and colleagues	Promising results in treating advanced melanoma	Eur. J Cancer 38(S7):74, 2002
2003	New approach to vaccination against cancer	Parish	Approach is potentially less susceptible to immune evasion	Immunology and Cell Biology 81, 106-113, 2003.

It can be argued that JCSMR's position as one of the original block-funded research schools of the Institute of Advances Studies (IAS) has given it the opportunity to pursue long-term, independent medical research. Many staff and alumni of JCSMR, including its present Director, argue that the Nobel Prizes, the Albert Einstein World Award, the Japan Prize, the Copley Medal, and a host of other international awards attest to the success of this funding strategy over the past 50 years, and that the scientific achievements that those prizes signify could only have been made by research that could be conducted over long (10 to 20 year) time frames.

But the funding climate has changed dramatically over the last few decades. Twenty five years ago many tenured Australian university or teaching hospital staff were able to undertake research even without external funding. Infrastructure was relatively generous, there was still funding for university technical research staff, hospitals provided drugs and consumables and even beds at no cost to the researcher, and research activities merged relatively seamlessly into those of teaching and patient care. Today research is increasingly separated from teaching and care, and the days of in-kind support are long gone. Research is an investment for the nation and the future, but in contemporary funding climates within universities and hospitals it is increasingly becoming an optional extra, undertaken only when researchers are able to attract sufficient external resources.

The change in environment and the disappearance of cross subsidisation from patient care and/or teaching has both advantages and disadvantages. Today's research (and research funding) is more transparent: what is spent on research as opposed to care and teaching, at least at the micro level, is much better defined. And, in parallel, the loss of capacity to undertake research that is not peer reviewed has almost certainly raised research standards. The negatives are equally obvious. The University of Melbourne submission to the Wills review stated 'The danger is that if the importance of the nexus between research and learning is not visible to tertiary students because University research is allowed to run down through poor infrastructure, equipment or lack of opportunity of research scientists then research institutes and teaching hospitals will inevitably suffer from a lack of quality of graduates available to them'[3].

The JCSMR has not been exempted from these pressures, and over the last 20 years the value of the block grant has been progressively eroded. The JCSMR grant was a one-line grant to the Director, which provided flexibility, but inhibited collaboration because of restrictive rules around the National Competitive Grants Scheme. Accordingly, a desire for transparency, competitiveness and collaboration led to a decision in 2000 by the Commonwealth Government that the IAS of the ANU could enter the National Competitive Grants Scheme. The negotiations that led to partial entry of JCSMR into the NHMRC schemes in 2001 (for 2002) were long and complex. However the negotiations between JCSMR and NHMRC were paralleled by those between the ARC and ANU and led to a jointly agreed 'buy in' price of 20% of 2000 funding for the IAS to gain access to the various national funding, research training and related schemes. This was $1.7 million per annum for JCSMR access to the National Competitive Grant Schemes and a similar amount for access to the training and infrastructure schemes. It was determined initially that entry would be phased, but after one year the ARC determined that the phase-in was unnecessary and gave the IAS full entry from 2002 (for 2003), and the NHMRC followed suit.

The JCSMR felt that, at last, it was able to redress the competitive disadvantage the School faced because of its essentially fixed funding over the last 10 to 15 years, at a time when government funding for the NHMRC system had doubled and then redoubled, from around $65 million in the late eighties to $176 million in 1999 to $381 million in 2004. The rest of the system waited, not without apprehension, for the outcome, but there had long been a wish within the research community that JCSMR be subject to the same forms of peer review as the broader medical research community.

Removing barriers to collaboration, the outcome of these changes, is important for 21^st ^century approaches to improving health. The World Health Organisation has stated that the likely trends in global health in the 21^st ^century include widespread absolute and relative poverty, demographic changes, ageing, growth of cities, epidemiological changes, continuing high influence of infectious diseases, increasing incidence of non-communicable diseases, injuries and violence, global environmental threats to human survival, new technologies, information and telemedicine services, advances in biotechnology, evolving partnerships for health that include private and public sectors and civil society, and globalisation of trade, travel and the spread of values and ideas. Research to deal with global health problems will therefore necessarily be multidisciplinary, involving biomedical, clinical, public health and health services research, and include the social sciences, information sciences and engineering, physics, chemistry, ecology and environmental sciences and economics. As part of the IAS, the JCSMR is accordingly strongly positioned. Not that this need for a multidisciplinary approach is really a new concept – in 1902 Osler stated that the remit of medical research was 'to wrest from nature the secrets which have perplexed philosophers in all ages, to trace to their sources the causes of disease, to correlate the vast stores of knowledge, that they may be quickly available for the prevention and the cure of disease – these are our ambitions' [4].

In the first three years that JCSMR has been eligible to apply for research funding, their researchers have been awarded a total of $18 million (88% NHMRC, 12% ARC) in competitive funding for periods of up to five years. All three NHMRC program grants held by researchers at the JCSMR are collaborative with other Australian institutions and School researchers hold four ARC linkage grants. This collaboration bodes well for Australian health and for biotechnology growth. In a highly competitive world, Australian researchers need every opportunity to succeed.
